# Orchestrating Cellular Balance: ncRNAs and RNA Interactions at the Dominant of Autophagy Regulation in Cancer

**DOI:** 10.3390/ijms25031561

**Published:** 2024-01-26

**Authors:** Xueni Yang, Shizheng Xiong, Xinmiao Zhao, Jiaming Jin, Xinbing Yang, Yajing Du, Linjie Zhao, Zhiheng He, Chengjun Gong, Li Guo, Tingming Liang

**Affiliations:** 1 State Key Laboratory of Organic Electronics and Information Displays & Institute of Advanced Materials (IAM), Nanjing University of Posts & Telecommunications, Nanjing 210023, China; 1022173309@njupt.edu.cn (X.Y.); 1022173308@njupt.edu.cn (S.X.); 1222014425@njupt.edu.cn (X.Z.); 1222014424@njupt.edu.cn (J.J.); 1223014120@njupt.edu.cn (L.Z.); 1023173006@njupt.edu.cn (Z.H.);; 2 Jiangsu Key Laboratory for Molecular and Medical Biotechnology, School of Life Science, Nanjing Normal University, Nanjing 210023, China; 221202124@njnu.edu.cn (X.Y.); 221202100@njnu.edu.cn (Y.D.)

**Keywords:** autophagy, miRNA, lncRNA, circRNA, interaction, cancer

## Abstract

Autophagy, a complex and highly regulated cellular process, is critical for the maintenance of cellular homeostasis by lysosomal degradation of cellular debris, intracellular pathogens, and dysfunctional organelles. It has become an interesting and attractive topic in cancer because of its dual role as a tumor suppressor and cell survival mechanism. As a highly conserved pathway, autophagy is strictly regulated by diverse non-coding RNAs (ncRNAs), ranging from short and flexible miRNAs to lncRNAs and even circRNAs, which largely contribute to autophagy regulatory networks via complex RNA interactions. The potential roles of RNA interactions during autophagy, especially in cancer procession and further anticancer treatment, will aid our understanding of related RNAs in autophagy in tumorigenesis and cancer treatment. Herein, we mainly summarized autophagy-related mRNAs and ncRNAs, also providing RNA–RNA interactions and their potential roles in cancer prognosis, which may deepen our understanding of the relationships between various RNAs during autophagy and provide new insights into autophagy-related therapeutic strategies in personalized medicine.

## 1. Introduction

The process of autophagy can effectively remove nutrients from cells and has been shown to inhibit cancer development [1,2]. By removing damaged proteins and organelles, autophagy can also limit oxidative stress and suppress oncogenic signals, further highlighting its potential significance in cancer [3,4,5,6,7]. Autophagy can have both positive and negative effects on the development of tumors, promoting the death of tumor cells and preventing the occurrence of tumors. It can also provide energy for cancer cells when they are under stress [8]. However, as the tumor progresses, tumor cells utilize autophagy in diverse ways to combat nutrient scarcity and hypoxia [9]. Regulating autophagy can promote cancer cell proliferation, but it can also help inhibit the expression of oncogenes. Decreased and abnormal autophagy inhibits the degradation of damaged components or proteins in oxidative stress cells, leading to cancer development [10]. Some autophagy-related genes (ATGs), such as *ATG2B, ATG5, ATG9B,* and *ATG12,* have been reported to contain frameshift mutations in cancer [11,12], implicating the potential contributions to tumorigenesis and cancer metastasis.

Both autophagy-related genes and non-coding RNAs (ncRNAs) are involved in the autophagy process, mainly including microRNAs (miRNAs), long non-coding RNAs (lncRNAs), and circular RNAs (circRNAs). Some genes have been used to treat patients in dozens of clinical trials aimed at regulating autophagy to treat or prevent diseases [13]. By directly suppressing *DACT3* in cancer cells, miR-638 can promote autophagy and malignant phenotypes [14], lncRNA MALAT1 may contribute to gastric cancer progression via inhibiting autophagic flux [15], and exosomal circ_0091741 can promote cell autophagy through the miR-330-3p/TRIM14/DvI2/Wnt/beta-catenin axis [16]. Some ncRNAs have been observed to participate in the regulation of autophagy, either by inducing or inhibiting it, leading to the modulation of cancer [17]. These diverse ncRNAs play a key role in cancer cell homeostasis and cancer progression by regulating autophagy. Clarifying the relationship between autophagy and ncRNAs will contribute to elucidating a promising potential therapeutic target in cancer treatment. Indeed, different types of ncRNAs can either promote or inhibit autophagy, which may, in turn, affect the migration of cancer cells [18]. The discovery of ncRNAs in autophagy has opened up new possibilities for understanding important biological processes. The expression and potential biological roles of ncRNAs have a significant impact on the level of cellular autophagy during different physiological and pathological stages, which may contribute to providing new insights for the diagnosis and treatment of cancer.

In order to shed light on the intricate relationships between autophagy-related genes, ncRNAs, and their potential impact on disease pathology, we mainly summarized autophagy-related pathways and RNAs, together with their biological function in pathological processes, especially the potential roles in cancer prognosis and treatment. Then, RNA interactions were further discussed, aiming to understand the possible regulatory network between various RNAs during autophagy, as well as their potential roles in autophagy-related therapeutic strategies. A profound understanding of the autophagy-related RNA regulatory network may contribute to clinical application in cancer diagnosis, classification, and treatment.

## 2. Autophagy-Related Genes and Cancer

**Figure 1:**
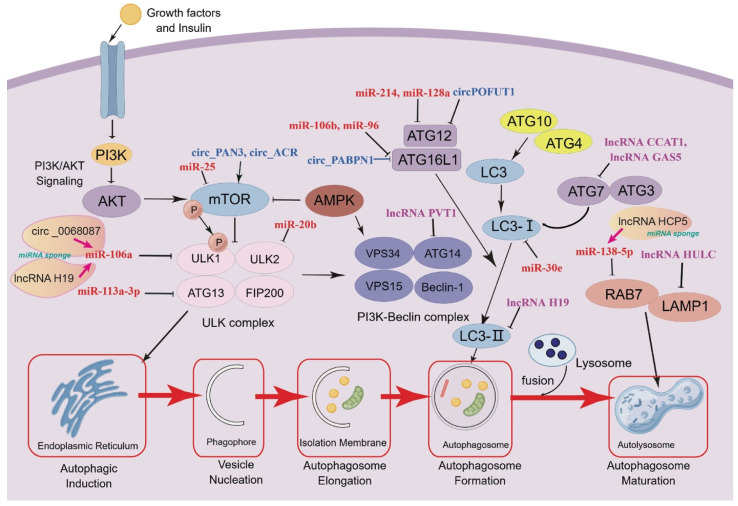
A simple model contains some autophagy-related genes and ncRNAs using Figdraw to indicate the interactions of autophagy-related genes and ncRNAs. Autophagy-related genes can be regulated by ncRNAs, and some lncRNAs and circRNAs may act as miRNA sponges to perturb gene expression.

Autophagy is typically divided into distinct stages: initiation, vesicle nucleation, vesicle elongation, vesicle fusion, and cargo degradation [19] (Figure 1). The first stage involves the induction of autophagy under stress, such as starvation and hypoxia. In the second stage, the PI3K complex initiates vesicle nucleation, and the third stage consists of autophagic membrane elongation and completion, which are regulated by two systems, the ATG12-ATG5-ATG16L and ATG8 (MAP1LC3 or LC3 in mammals) systems [20]. The autophagosome fuses with the lysosome and is then degraded, and the macromolecules are reused to fuel relevant metabolic pathways. The biological process of autophagy serves a vital function in breaking down proteins and organelles to prevent the buildup of harmful waste and maintain the proper functioning of cells and organisms [21,22]. It effectively removes misfolded proteins and damaged organelles [23] and can promote survival in nutrient-deficient environments [24]. Without its crucial role, abnormal cell function, reactive oxygen species (ROS) imbalances, inflammation, and antigen presentation defects could occur [25]. Thus, the cells are prone to malignant transformation into tumor cells. Autophagy plays an important role in both tumor suppression and tumor promotion, and *mice* with systemic mosaic deletion of *Atg5* and liver-specific *Atg7^−/−^* can develop benign liver adenomas [26].

It is generally accepted that autophagy can inhibit the growth and development of tumor cells. In some cases, autophagy can promote tumor suppression by removing specific factors, such as *p62* and *p53*, and elevated levels of *p62*, found in many cancer types, are thought to promote tumors [27], while the deficiency of *p53* accelerates pancreatic tumor progression [28]. Some genes are identified as autophagy-related genes at different stages (Table 1), such as the MTORC1 protein, which senses nutrients acting as a suppressor of autophagy. NCAPD2 can restrict autophagy by regulating the Ca^2+^/CAMKK2/AMPK/mTORC1 pathway, thereby promoting colorectal cancer [29]. Meanwhile, *AMPK* is activated during instances of energy deprivation and encourages autophagy, which fosters the formation of dormant polyploid giant cancer cells [30]. Autophagy is heightened in hypoxic regions of tumors, which are vital for the survival of cancer cells. The removal of the BECN1 gene (*Beclin-1*) increases the likelihood of postpartum breast tumor occurrence [31]. The deliberate suppression or removal of critical autophagy genes in cancer cells has been shown to decrease their ability to survive and form tumors, and the activation of tumor pathways and stress in the tumor microenvironment may increase the need for autophagy to aid in tumor growth and survival. Based on its critical roles in multiple biological processes, autophagy has a global role in metabolism, protein and organelle quality control, and the relationship between autophagy and anti-tumor immune response will enrich the relevant studies, especially in cancer treatment. A better understanding of how cancer cells overcome the inhibitory effects of autophagy to progress, as well as how autophagy maintains established survival, is crucial. The regulation of autophagy is quite complex in tumorigenesis and cancer progression, and the detailed molecular mechanism and possible clinical application in anticancer therapeutic strategies remain a challenge.

**Figure 2:**
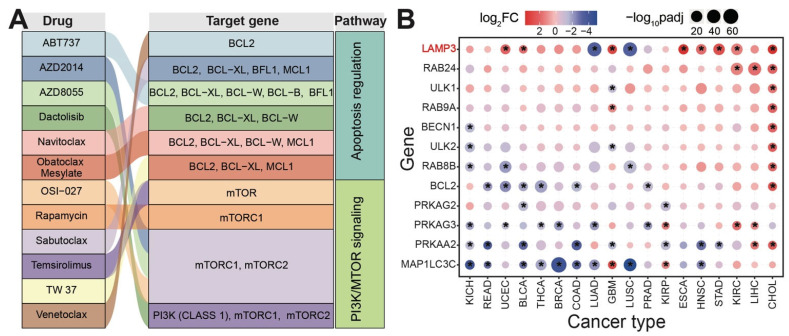

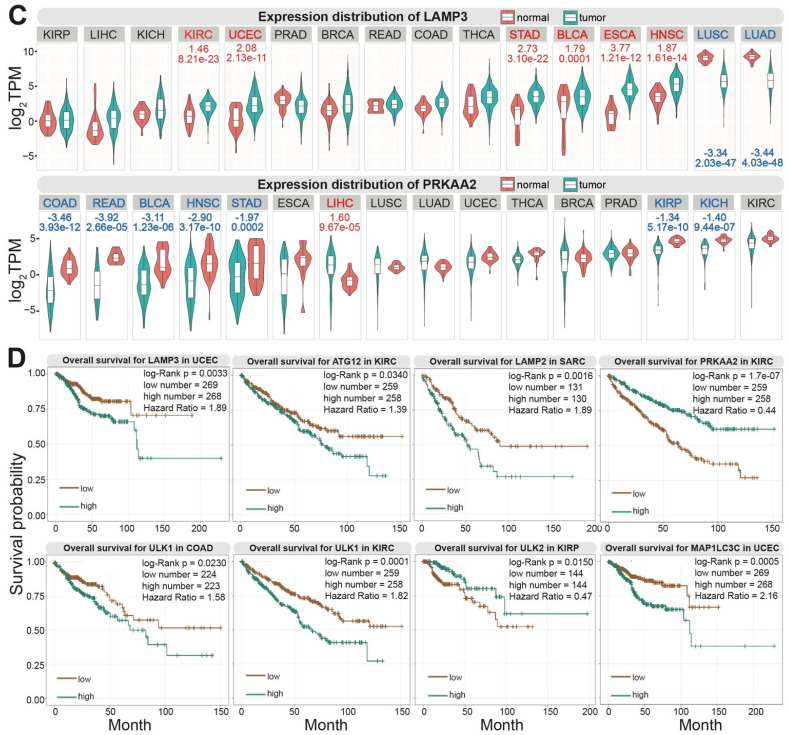
Autophagy-related genes may be drug targets and have potential value in cancer prognosis and treatment. (**A**). Some autophagy-related genes are potential drug targets that are mainly involved in apoptosis regulation and PI3K/MTOR signaling pathways. The relationships among drugs, genes, and pathways were calculated with the oncoPredict R package [40] based on Genomics of Drug Sensitivity in Cancer (GDSC) data [41]. (**B**). Some autophagy-related genes indicate diverse expression distributions across different cancers according to sequencing data in The Cancer Genome Atlas (TCGA). * indicates significantly upregulated or downregulated in specific cancer (|log_2_FC| > 1.2 and padj < 0.05), using the limma package [42]. Abbreviations of cancers in (**B**): BLCA, bladder urothelial carcinoma; BRCA, breast invasive carcinoma; CHOL, cholangiocarcinoma; COAD, colon adenocarcinoma; ESCA, esophageal carcinoma; GBM, glioblastoma multiforme; HNSC, head and neck squamous cell carcinoma; KICH, kidney chromophobe; KIRC, Kidney renal clear cell carcinoma; KIRP, kidney renal papillary cell carcinoma; LIHC, liver hepatocellular carcinoma; LUAD, lung adenocarcinoma; LUSC, lung squamous cell carcinoma; PRAD, prostate adenocarcinoma; READ, rectum adenocarcinoma; STAD, stomach adenocarcinoma; THCA, thyroid carcinoma; UCEC, uterine corpus endometrial carcinoma. (**C**). Examples of the detailed expression distributions of autophagy-related genes across different cancers. For significantly upregulated or downregulated expression, log_2_FC and padj are also presented (red shows upregulated expression; blue shows downregulated expression). (**D**). Survival analysis (according to the starBase database [43]) shows that some genes may be associated with cancer prognoses.

As a natural and complex cellular process, multiple signaling pathways are involved in the physiological process of autophagy, such as the PI3K-AKT-mTOR and MAPK-Erk1/2 pathways. The PI3K/protein kinase B(*AKT*)/mTOR signaling pathway, which inhibits autophagy in conditions of nutrient enrichment, is activated when PI3K binds to growth factor receptors. AKT is activated, in turn activating mTOR. However, PTEN can antagonize PI3K activity, thereby inhibiting AKT activity and mTOR activation, and then inducing autophagy [32]. Upstream signals of the autophagy signaling pathway are mainly involved in the mammalian target of the rapamycin (mTOR)-dependent pathway and mTOR-independent pathway, such as AMP-activated protein kinase (AMPK), PI3K, Ras-MAPK, p53, PTEN, and endoplasmic reticulum stress [33]. The process of autophagy induction relies heavily on mTOR kinase. Autophagy is inhibited by the activation of mTOR pathways, such as the AKT, MAPK, PI3K-I/Akt and MAPK/Erk1/2 signaling pathways, while it is promoted by the negative regulation of mTOR pathways. ULK is a key autophagy core protein with serine/threonine kinase activity. The activation of the ULK complex in autophagic signaling, including ULK1 or ULK2, FIP200, and ATG13, occurs prior to autolysosome assembly [34]. The ULK1 complex serves as a bridge in vivo, connecting the upstream nutrient or energy sensors mTOR and AMPK with the formation of downstream autophagosomes. The phosphorylation of ULK1 has long been recognized as a critical regulator of autophagy. Recently, two kinases, AMPK and mTOR, have been discovered to catalyze the phosphorylation of ULK1 [35], which may play a pivotal role in autophagy. In the presence of adequate nutrition, when AMPK is inactivated, mTOR can bind to ULK1 serine 757, leading to the inhibition of ULK1–AMPK interaction, inactivation of ULK1, and, ultimately, the cessation of autophagy signaling. There are both positive and negative links between apoptosis and autophagy, and there is extensive signal “talk” between the two processes. When nutrients are deficient, autophagy functions to promote cell survival, but excessive autophagy can lead to autophagic cell death, which is morphologically distinct from apoptosis. Autophagy-related genes may be potential drug targets via involvement in apoptosis regulation and PI3K/MTOR signaling pathways (Figure 2A) [36], implying their potential clinical application in cancer treatment [37]. These genes always show dynamic expression patterns in different cancer types (Figure 2B,C), and abnormal expression patterns imply that these genes may be critical in cancer tumorigenesis and metastasis [38]. For example, LAMP3 is detected with higher expression pattern in some cancers, and overexpression may play a role in tumorigenesis. Patients with higher or lower expression of specific genes may have better survival (Figure 2D), suggesting potential roles for these genes in cancer prognosis [39].

## 3. Regulation Roles of ncRNAs in Autophagy

Many autophagy-related genes may be directly or indirectly regulated by diverse ncRNAs (Figure 1), demonstrating complex RNA interactions during the essential stages of autophagy, including autophagy initiation, vesicle nucleation, autophagosome elongation, autophagosome formation, and maturation [10]. They are also involved in regulating the upstream signaling pathways that control autophagy induction. Some ncRNAs can directly regulate genes related to autophagy and are known to have significant impacts on various stages of the process and cancer development. Numerous human diseases, ranging from cancer and neurodegeneration to metabolic disorders such as diabetes and organ-related ailments such as heart, lung, liver, kidney, and stomach issues, have been linked to autophagy dysfunction that may be involved in the regulatory roles of ncRNAs (Table 2, Table 3 and Table 4).

### 3.1. Regulation of ncRNAs in Autophagy Initiation

Autophagy in higher mammals is mainly triggered by ULK complexes and facilitated by AMPK, AKT, mTOR, ULK complex, etc. NcRNAs primarily control the process of autophagy induction by regulating these compounds, such as factors that affect cancer cell migration. Understanding the role of ncRNAs in regulating autophagy in diseases will provide new strategies for the clinical treatment of various autophagy-related diseases.

NcRNAs can influence the initiation phase of autophagy in human cancer by regulating the expression of various components in the ULK complex, which is a key autophagy core protein during the initiation of autophagy. For example, miR-17 family members, miR-20a and miR-106b, may regulate autophagy induced by leucine deprivation in C2C12 myoblasts by inhibiting *ULK1* expression [53]. It has been confirmed that other members of the miR-17 family, namely miR-20b, miR-106a, miR-93, and miR-17-5p, also inhibit the expression of *ULK1*, which in turn inhibits autophagy [44]. Autophagy protects lung adenocarcinoma cells by decreasing ULK1 expression via the miR-106a-ULK1 axis [45]. Other miRNAs, including miR-489, miR-142-5p, and miR-25, can affect autophagy by targeting *ULK1* [46,47,48]. ULK2 is regulated by miR-885-3p, suggesting that miR-885-3p might contribute to the regulation of squamous cell carcinoma cell autophagy and/or apoptosis upon cisplatin exposure [51]. Furthermore, lncRNA SNHG6 is able to promote colorectal cancer chemoresistance and enhance autophagy through regulation of *ULK1* by sponging miR-26a-5p [79]. Knockdown of lncRNA AK044604 (regulator of insulin sensitivity and autophagy, RISA), a regulatory factor, regulates insulin sensitivity and autophagy in *mice*, increases the phosphorylation of ULK1, and thus helps initiate autophagy and weaken insulin resistance [106]. Circ_0009910 has been found to regulate the expression of *ULK1* by sponging miR-34a-5p in chronic myeloid leukemia, thereby activating the level of autophagy [96], and circ_CDYL accelerates autophagic flux via sponging miR-1275 and regulating the expression of autophagy-related genes *ATG7* and *ULK1*, thus promoting autophagy and the progression of breast cancer [97]. *ATG13* can be regulated by miR-133a-3p, and *FIP200* can be simultaneously regulated by several miRNAs, including miR-20a, miR-20b, miR-224-3p, and miR-309-3p [52]. In addition, circMUC16 can directly associate with ATG13, stabilize its expression, and then promote autophagy in epithelial ovarian cancer by regulating *Beclin1*, *RUNX1*, and *ATG13* [101].

mTOR plays an important role in the initiation of cell autophagy and can be promoted via activated AKT. The miR-99 family, comprising miR-99a, miR-99b, and miR-100, can indirectly promote autophagy by inhibiting the IGF-1R/AKT/mTOR signaling pathway, while miR-100 can inhibit mTOR and then activate autophagy [54]. miR-378 promotes autophagy initiation through the mTOR/ULK1 axis and sustains autophagy via FoxO-mediated transcriptional reinforcement [76]. LncRNAs can also regulate autophagy by directly or indirectly affecting mTOR molecules. For example, overexpression of *NBR2* can inhibit the mTOR pathway and AMPK is activated, boosting AMPK levels in colorectal cancer under energy stress [77]. LncRNA H9 plays an important role in p38/AMPK/mTOR, toll-like receptor, and autophagic activation [107], and overexpression of lncRNA PTENP1 indirectly inhibits the PI3K/AKT pathway through *PTEN* overexpression and then induces pro-death autophagy, leading to the death of hepatocellular carcinoma cells [84,85]. In esophageal squamous cell carcinoma, circRNA ciRS-7 affects the epidermal growth factor receptor AKT-mTOR signaling pathway, thus inhibiting the autophagy of ESCC cells [100].

### 3.2. Regulation of ncRNAs in Vesicle Nucleation

During vesicle nucleation, proteins and liposomes combine to form double-membrane binding vesicles, known as autophagosomes. This process is mainly initiated by a complex of autophagy-related proteins called the class III phosphatidylinositol 3-kinase (PI3K) complex. Diverse ncRNAs have powerful regulatory versus control roles during the vesicle nucleation stage.

*Beclin-1*, one of the key molecules of autophagosome nucleation, is a critical target for regulating autophagy, and may play a key role in whether cells eventually go to autophagy or apoptosis. Several miRNAs, including miR-124-3p, miR-216b, miR-376b, miR-409-3p, and members of the miR-30 family, have been reported to affect the expression of *Beclin-1* and autophagy by targeting the 3′-UTR of *Beclin-1* [58]. miR-30a targets *Beclin-1*, which mediates autophagy [58], and inhibits autophagy by downregulating the expression of *Beclin-1* in medulloblastoma [59]. miR-216b can inhibit cisplatin sensitivity of non-small cell lung cancer through regulating apoptosis and autophagy via miR-216b/Beclin-1 pathway [63], and miR-143 plays an essential role in tumorigenesis and chemotherapy resistance by targeting the various cellular and molecular pathways (i.e., PI3K/AKT/Wnt, EMT, p53, and ATM) involved in the autophagy pathways pathogenesis of colorectal cancer [65]. Downregulation of lncRNA MALAT1 attenuates neuronal cell death through suppressing *Beclin1*-dependent autophagy by regulating miR-30a expression in cerebral ischemic stroke [108]. The expression of lncRNA SNHG12 is upregulated in mouse MCAO models and OGD/R models in SH-SY5Y cells, promoting *LC3-II* and *Beclin-1* expression levels and thus inducing autophagy activation [86]. CircRNF144B promotes the ubiquitination of *Beclin-1* by sponging injection of miR-11-342p, thereby inhibiting autophagic flux and promoting ovarian cancer progression [109]. In epithelial ovarian cancer, circMUC16 can promote the expression of *Beclin-1* and *Runx1* by sponging miR-199a-5p, thus promoting autophagy [101].

*ATG7, ATG14,* and the *Vps34* complex also play an important role in the process of autophagosome nucleation. Overexpression of lncRNA PVT1 increases the expression levels of *ATG7*, which is essential for autophagy initiation and the formation of a double-membrane structure, thus inducing autophagy [110]. LncRNA BCRP3 is a positive regulator of autophagy, mostly found in the cytoplasm, which binds to the Vps34 complex to enhance its enzymatic activity [111]. *PVT1* interacts with *ATG14* in the cytoplasm and can upregulate the expression of *ATG14* and thus regulate autophagic activity [88].

### 3.3. Regulation of ncRNAs in Autophagic Vesicle Elongation

*ATG12* is driven by *ATG7* (an E1-like enzyme) and *ATG10* (an E2-like enzyme), conjugates with *ATG5*, and then interacts with *ATG16* (mammalian orthologous ATG16L) to form the ATG12–ATG5–ATG16 complex. *LC3* is then converted from its cytoplasmic-soluble isoform (LC3-I) to its membrane-anchored isoform (LC3-II) by the ATG12-ATG5-ATG16 complex, together with *ATG7* and *ATG3* (an E2-like enzyme). *ATG12*, *ATG5*, and *ATG16* participate in the elongation of the autophagic vesicle [112].

miR-214 significantly increases the radiosensitivity of colorectal cancer via the inhibition of autophagy and induction of apoptosis by targeting *ATG12* [71]. *ATG12* is also a target of circPOFUT1 in regulating autophagy-related chemical resistance, and circPOFUT1 promotes *ATG12* expression to regulate autophagy-associated chemoresistance by sponging miR-488-3p in gastric cancer [102]. miR-106a and miR-106b, two members of the miR-17 family, have been shown to inhibit starvation-induced autophagy in colorectal cancer cells, while only miR-106b inhibits starvation-induced autophagy by inhibiting the expression of *ATG16L1* [72]. Autophagy inhibition occurs when important genes, such as *ATG16L1* and *ATG12*, are targeted. LncRNA CCAT1 facilitates hepatocellular carcinoma cell autophagy and cell proliferation by functioning as a sponge for miR-181a-5p and then regulating *ATG7* expression [91]. Interference with lncRNA SNHG3 improves brain I/R injury by downregulating *ATG7* to restrain autophagy [113]. The inflammation-induced ectopic expression of lncRNA TGFB2-OT1 activates autophagy via increasing the expression levels of *ATG13*, *ATG3*, *ATG7*, and *P62,* and the small molecule inhibitor 3BDO significantly decreases *TGFB2-OT1* levels and inhibits subsequent autophagy and inflammation [114]. LncRNA HNF1A-AS1, sponging miR-30b from binding to its target of *ATG5*, provokes autophagy in hepatocellular carcinoma. Moreover, *Beclin-1* and *ATG12* have also been defined as targets of miR-30b, indicating that HNF1A-AS1 upregulates *Beclin-1*, *ATG5* and *ATG12* expression to promote elongation of the autophagic vesicle [93,115]. Circ_0092276 can repress *ATG7* via sponging miR-384, thus effecting autophagy and proliferation as well as repressing apoptosis of breast cancer cells [103].

During autophagic vesicle elongation, the LC3 protein is cleaved by *ATG4* at its carboxyl terminus immediately after synthesis, resulting in the production of LC3-I localized in the cytoplasm. LncRNA NEAT1 can induce abnormal autophagy by stabilizing *PINK1*, which is an LC3-II upstream regulatory factor and plays a role in the pathogenesis of Parkinson’s disease [90]. In epithelial ovarian carcinoma, overexpression of HULC reduces *ATG7*, *LC3-II* and *LAMP1* expression while inducing SQSTM1 (P62) and ITGB1 expression, thus inducing cell proliferation, reducing apoptosis, and inhibiting autophagy in vitro [95]. In drug-resistant renal clear cell carcinoma cells treated with gemcitabine, when circ_0035483 expression is downregulated, the *LC3-II/LC3-I* ratio is significantly reduced, thus inhibiting autophagy [104]. Hsa_circ_0092276 overexpression effects the proliferation of breast cancer cells, while hsa_circ_0092276 silencing represses the expression of *LC3-II/LC3-I* and *Beclin-1* [103]. These diverse ncRNAs contribute to autophagic vesicle elongation via direct or indirect interactions with critical autophagy-related genes, and their important regulatory roles also provide the possibility of discovering potential drug targets in cancer treatment.

### 3.4. Regulation of ncRNAs in Autophagosome Formation and Maturation

*ATG7* and *ATG16L1* also play important roles in the process of autophagosome formation. They are upregulated in the neurons and promote autophagosome formation. Overexpression of miR-96 significantly prevents brain damage in SE rats by inhibiting *ATG7* and *ATG16L1* expression and autophagosome formation in the hippocampus [116]. Circ_PABPN1 competitively binds HuR, blocks its binding to *ATG16L1*, inhibits *ATG16L1* translation, and thus regulates autophagy in intestinal epithelial cells [105]. In intestinal epithelial cells, the targeted deletion of HuR specifically reduces the level of *ATG16L1* in the intestinal mucosa, while circPABPN1 can bind to HuR to enhance autophagy [105]. LncRNA 17A knockdown increases the expression levels of *LC3-II,* which is a hallmark of autophagosome formation [117]. The expression of *LC3*, *P62*, and *LAMP2* can be regulated by lncRNA MALAT1, which represses autolysosome fusion via the downregulation of *LAMP1* and *LAMP2*, leading to autophagic inhibition [118].

The autophagosome maturation process requires several complexes, including integral lysosomal proteins (such as LAMP1, LAMP2, and LAMP3) and RAB proteins (such as RAB5 and RAB7), to aid in autophagosome–lysosome fusion and maturation [119,120]. miR-138-5p contributes to this process via targeting *SIRT1* to inhibit autophagy in pancreatic cancer by indirectly regulating *RAB7* [73]. miR-487b-5p directly targets *LAMP2*, affecting autophagy in cortical neurons [74], and miR-207 and miR-352 can affect autophagy by directly targeting *LAMP2* in ischemic stroke [121]. miR-224, miR-21, miR-373-5p, and miR-379 can interact with *LAMP2*, regulating its expression [122,123]. Additionally, miR-205 inhibits autophagy by targeting *RAB27A* and *LAMP3*, leading to increased cisplatin cytotoxicity in prostate cancer cells [75]. Certain miRNAs are capable of targeting multiple proteins at different stages of autophagy. Specifically, miR-33a-5p and miR-33a-3p can directly target *ATG5*, *ATG12*, *LC3B*, and *LAMP1* [124], and these miRNAs can also inhibit AMPK-dependent autophagic activation and lysosomal gene transcription by targeting *FOXO3* and *TFEB*. Hsa_circ_0001658 suppresses the autophagy of gastric cancer cells via the miR-182/RAB10 axis and sponges miR-182 to suppress the expression of *RAB10* [125].

**Figure 3:**
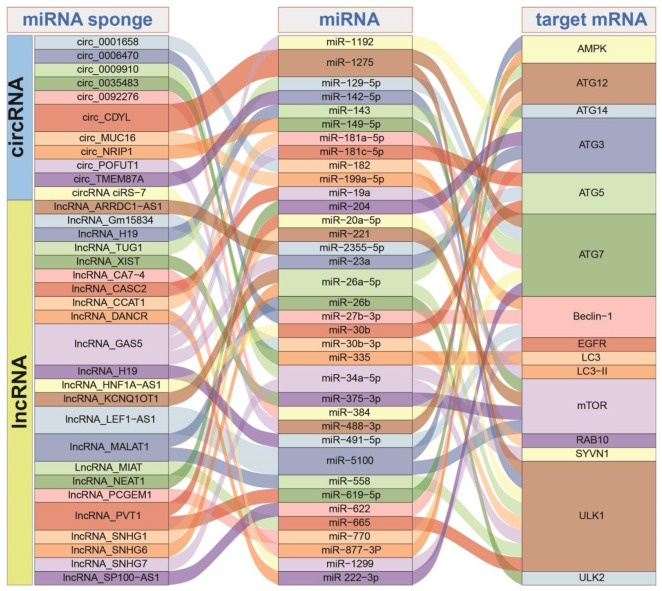
An example of autophagy-related RNA interactions based on ceRNA regulatory networks. Some lncRNAs and circRNAs have been reported as miRNA sponges to perturb the expression levels of autophagy-related genes.

Taken together, diverse ncRNAs have been found to play a significant role in the autophagy process, which involves the breakdown and recycling of cellular components, implicating the potential complex interaction network among different RNAs, especially via ceRNA networks (Figure 3). Some lncRNAs and circRNAs may act as miRNA sponges to perturb the miRNA regulatory network and then disturb the expression levels of target mRNAs, although many ncRNAs also can regulate mRNA expression via binding target mRNAs as important regulators. As a critical interaction method, ceRNA networks have been widely studied because the RNA interactions among different RNAs may contribute to multiple biological processes, even in tumorigenesis. According to autophagy-related RNAs, several genes are involved in different ceRNA networks, such as ATG7, mTOR, and ULK1 (Figure 3), indicating that these autophagy-related genes are prone to be strictly regulated by multiple ncRNAs via a complex RNA interaction. The dysregulation of ncRNAs or genes may contribute to metabolic disorders, neurodegenerative disorders, and cancer. The intricate relationships between RNAs during autophagy should be further considered to reveal their potential roles in cancer prognosis and treatment.

## 4. Conclusions and Perspective

Autophagy plays a dual role in cancer, and it is crucial to gain a better understanding of how tumors overcome autophagy’s growth-inhibiting effects to promote tumor development while maintaining or restoring autophagy to sustain established tumors. Diverse ncRNAs contribute to autophagy as regulators via RNA interactions, especially via a ceRNA regulatory network that has been widely considered as a potential biomarker for cancer diagnosis and prognosis. As a class of pivotal regulators, ncRNAs have spatiotemporal specificity and tissue specificity, indicating that they may be potential biomarkers and therapeutic targets for autophagy-related diseases. Based on the important regulatory roles of ncRNAs in a coding-non-coding RNA interaction network, the regulatory network containing various RNAs is more complex than we previously thought, and the detailed interaction mechanism may provide novel strategies for autophagy-related diseases, particularly for cancer. Although many ncRNAs have been reported as critical regulators, more ncRNAs may contribute to autophagy process via direct or indirect interactions with autophagy-related genes, and the detailed ncRNA–mRNA interaction profile should be explored to systematically understand autophagy-associated regulatory networks. It is encouraging that the interactions of different autophagy-associated RNAs may be combined with traditional chemotherapy or anti-tumor immune response strategies to potentially benefit cancer patients.

## Figures and Tables

**Table 1:** Some related genes at different stages of autophagy.

Autophagy Stage	Name	Composition
Initiation	Energy depletion	AMPK
	mTOR complex	mTORC1
		mTORC2
	Class III PI3K complex	Vps34
		Vps15
	ULK complexes	FIP200
		ULK1
		ULK2
		ATG13
		ATG101
	Class II PI3K complex	Beclin-1
Vesicle nucleation	Class III PI3K complex	Vps34
		Vps15
	Class II PI3K complex	Beclin-1
	Bcl-2 family	Bcl-2
	-	ATG14
Vesicle elongation	Atg12-Atg5-Atg16	ATG7
		ATG5
		ATG12
		ATG16
	LC3	LC3-I
		LC3-II
		ATG4B
Autophagosome formation (Vesicle fusion)		STX17
	-	ATG10
	LC3	LC3-I
		LC3-II
	-	Rab7
	-	Rab5
	-	Rab9
Maturation and degradation	-	p62
	LC3	LC3II
	-	Rab7
	-	Rab8B
	-	Rab24
	LAMP	LAMP1
		LAMP2
		LAMP3

**Table 2:** Some related miRNAs at different stages of autophagy.

Autophagy Stage	miRNA	Disease/Cancer	Target	Function	Signaling Pathway/Axis	Ref.
initiation	miR-20a/20b	breast cancer	RB1CC1/FIP200	Overexpression of miR-20a and miR-20b attenuates autophagy	-	[44]
initiation	miR-106a	lung adenocarcinoma	ULK1	miRNA-106a targeted ULK1 results in death of different NSCLC cells	miR-106a-ULK1	[45]
initiation	miR-489	breast cancer	ULK1, LAPTM4B	miR-489 affects autophagy by targeting ULK1	-	[46]
initiation	miR-25	breast cancer	ULK1	miR-25 functions as a regulator of autophagy by targeting ULK1	-	[47]
initiation	miR-142-5p	gastric cancer	ULK1	miR-142-5p can regulate ULK1 expression	-	[48]
initiation	miR-26	non-small cell lung cancer	TGF-β1	miR-26 reduces autophagy via targeting TGF-β1	TGF-β1-JNK	
initiation	miR-17-5p	cells	ULK1	miR-17-5p inhibits ULK1 expression in cellular autophagy	-	[49]
initiation	miR-26b	breast cancer	DRAM1	miR-26b can suppress autophagy in breast cancer cells	TGF-β1-JNK	[50]
initiation	miR-885-3p	squamous cell carcinoma	ULK2	miR-885-3p contributes to the regulation of squamous cell carcinoma cell autophagy	-	[51]
initiation	miR-133a-3p	gastric cancer	ATG13, GABARAPL1	miR-133a-3p expression inhibits autophagy to hinder gastric cancer metastasis via blocking GABARAPL1 and ATG13 expression	-	[52]
initiation	miR-20a	C2C12 myoblasts	ULK1	miR-20a inhibits the expression of ULK1, which leads to a reduction in autophagy induced by leucine deprivation	PI3K-AKT- MTOR	[53]
initiation	miR-100	renal cell carcinoma	mTOR	miR-100 can inactivate mTOR and thus increase autophagy in renal cancer cells.	mTOR	[54]
vesicle nucleation	miR-30a	hepatic fibrosis	Beclin-1	Overexpression of miR-30a inhibits Beclin1-mediated autophagy to prevent the occurrence of liver fibrosis	-	[55]
vesicle nucleation	miR-93	glioblastoma	BECN1, Beclin-1, ATG5, ATG4B, SQSTM1/p62	miR-93 inhibits autophagy functions by targeting multiple autophagy regulators	PI3K-AKT	[56]
vesicle nucleation	miR-124-3p	breast cancer	Beclin-1, LC3-I	miR-124-3p promotes the progression of breast cancer cells by enhancing the expression of Beclin-1	-	[57]
vesicle nucleation	miR-30a	cardiomyocyte	Beclin-1	Downregulation of miR-30a expression upregulates beclin-1 expression and enhances autophagy in cardiomyocytes	-	[58]
vesicle nucleation	miR-30a	medulloblastoma	Beclin-1, LC3B	miR-30a inhibits autophagy by downregulating the expression of Beclin-1and LC3B	-	[59]
vesicle nucleation	miR-30e	cardiomyopathy	Beclin-1, LC3-I, LC3-II	miR-30e can downregulate the expression of Beclin-1	-	[60]
vesicle nucleation	miR-30d	colon cancer	Beclin-1	Overexpression of miR-30d inhibits the proliferation of colon cancer cells	-	[61]
vesicle nucleation	miR-30d	renal cell carcinoma	MTDH	miR-30d targets MTDH and inhibits renal cancer cells	AKT/FOXO	[62]
vesicle nucleation	miR-124-3p	breast cancer	Beclin-1	Decreased miR-124-3p expression prompts breast cancer cell progression	-	[57]
vesicle nucleation	miR-216b	non-small cell lung cancer	Beclin-1	miR-216b can inhibit cisplatin sensitivity of NSCLC through regulating apoptosis and autophagy via miR-216b/Beclin-1 pathway	miR-216b/Beclin-1 axis	[63]
vesicle nucleation	miR-17-5p	non-small cell lung cancer	Beclin-1	miR-17-5p facilitates the ability of cell proliferation, inhibits autophagy and apoptosis by modulating Beclin-1	-	[64]
vesicle nucleation	miR-143	colorectal cancer	Beclin-1	miR-143 targets various cellular that are involved in the autophagy pathways pathogenesis of colorectal cancer	PI3K/AKT/Wnt	[65]
elongation	miR-23a	fibroblasts	AMBRA1	miR-23a inhibits the autophagy of fibroblasts during UV-induced photoaging	-	[66]
elongation	miR-23a-5p	acute myeloid leukemia	TLR2	Downregulation of miR-23a-5p in leukemic cells can lead to the upregulation of protective autophagy	-	[67]
elongation	miR-7	lung cancer	AMBRA1	AMBRA1 is targeted by miR-7, leading to the promotion of lung cancer cell proliferation	AKT	[68]
elongation	miR-128a	osteoarthritis	ATG12	ATG12, induced by miR-128a, loss represses chondrocyte autophagy to aggravate OA progression	-	[69]
elongation	miR-23b	traumatic brain injury	ATG12	miR-23b directly targets to the 3′UTR region of ATG12 to suppress the activation of neuronal autophagy	-	[70]
elongation	miR-214	colorectal cancer	ATG12, LC3	miR-214 inhibits autophagy and induction of apoptosis by targeting ATG12	-	[71]
autophagosome formation	miR-106b	colorectal cancer	ATG16L1	miR-106b inhibits starvation-induced autophagy by inhibiting the expression of ATG16L1	-	[72]
autophagosome maturation	miR-138-5p	pancreatic cancer	SIRT1	miR-138-5p specifically targets SIRT1, thereby inhibiting autophagy.	-	[73]
autophagosome maturation	miR-487b-5p	lung cancer	LAMP2	miR-487b-5p directly targets LAMP2 to affect the latter stage of autophagy flux in lung cancer	-	[74]
autophagosome maturation	miR-205	prostate cancer	RAB27A, LAMP3	miR-205 inhibits autophagy in prostate cancer cells	-	[75]
autophagosome maturation	miR-378	-	PDK1	miR-378 promotes autophagy initiation through the mammalian target of rapamycin mTOR/ULK1 pathway and sustains autophagy by targeting phosphoinositide-dependent protein kinase 1 (PDK1)	mTOR/ULK1	[76]

**Table 3:** Some related lncRNAs at different stages of autophagy.

Autophagy Stage	lncRNA	Cancer/Disease	Target	Function	Signaling Pathways/Axis	Refs.
initiation	lncRNA NBR2	colorectal cancer	AMPK	AMPK promotes the activation of autophagy by binding to lncRNA NBR2	mTOR	[77]
initiation	lncRNA AD5-A lncRNA	hepatocellular carcinoma (HCC)	AKT, mTOR	Overexpression of AD5-A lncRNA can block the function of miRNAs to inhibit AKT/mTOR activity and promote autophagy activation	AKT/mTOR	[78]
initiation	lncRNA SNHG6	colorectal cancer	ULK1	lncRNA SNHG6 is able to promote colorectal cancer chemoresistance and enhance autophagy through regulation of ULK1	-	[79]
initiation	lncRNA MALAT1	brain microvascular endothelial cell injury	ULK2	lncRNA MALAT1 can promote the expression of ULK2, suggesting that MALAT1 protects brain microvascular endothelial cells from ischemia-reperfusion injury by promoting autophagy	-	[80]
initiation	lncRNA H19	cardiomyocytes	DIRAS3	H19 could inhibit cardiomyocyte autophagy by epigenetically silencing DIRAS3	mTOR	[81]
initiation	lncRNA SNHG1	parkinson’s disease	LC3-II	Downregulated lncRNA SNHG1 inhibits the mTOR pathway and initiates autophagy	mTOR	[82]
initiation	lncRNA AK156230	mouse embryonic fibroblasts	mTOR	AK156230 can inhibit replicative senescence (RS); meanwhile, the mTOR signaling pathway leads to autophagy deficiency, which may accelerate aging	mTOR	[83]
initiation	lncRNA PTENP1	hepatocellular carcinoma cells	AKT	Overexpression of lncRNA PTENP1 indirectly inhibits the PI3K/AKT pathway and then induces pro-death autophagy, leading to the death of hepatocellular carcinoma cells	PI3K/AKT	[84,85]
vesicle nucleation	lncRNA SNHG12	SH-SY5Y cells	LC3-II, Beclin-1	The expression of lncRNA SNHG12 promotes LC3-II and Beclin-1 expression levels, thus inducing autophagy activation	-	[86]
vesicle nucleation	lncRNA AC023115.3	human glioblastoma cells	Beclin-1	AC023115.3 is induced by cisplatin, and elevated AC023115.3 promotes cisplatin-induced apoptosis by inhibiting autophagy	miR-26a-GSK3β-Mcl1 axis	[87]
vesicle nucleation	lncRNA PVT1	-	ATG14	PVT1 interacts with ATG14 in the cytoplasm, and PVT1 can upregulate the expression of both Pygo2 and ATG14, thus regulating autophagic activity	-	[88]
vesicle nucleation	lncRNA EIF3J-DT	gastric cancer	ATG14	EIF3J-DT activates autophagy and induces drug resistance in gastric cancer cells by targeting ATG14, thus contributing to activation of autophagy	-	[89]
vesicle nucleation	lncRNA NEAT1	Parkinson’s disease	LC3-II	lncRNA NEAT1 can induce abnormal autophagy by stabilizing PINK1, which is an LC3-II upstream regulatory factor and plays a role in the pathogenesis of PD	-	[90]
elongation	lncRNA CCAT1	hepatocellular carcinoma cell	ATG7	lncRNA CCAT1 facilitates hepatocellular carcinoma cell autophagy and cell proliferation, and then regulates ATG7 expression	-	[91]
elongation	lncRNA GAS5	osteoarthritis	Beclin-1, ATG3, ATG5, ATG7, ATG12	lncRNA GAS5, upregulating in osteoarthritis (OA), contributes to the pathogenesis of OA and thereby represses autophagy	-	[92]
elongation	lncRNA HNF1A-AS1	hepatocellular carcinoma	ATG5, Beclin-1, ATG12	lncRNA HNF1A-AS1, binding to its target Beclin-1, ATG5, and ATG12, can provoke autophagy in hepatocellular carcinoma	-	[93]
elongation	lncRNA HOTAIR	hepatocellular carcinoma	ATG3, ATG7	lncRNA HOTAIR is upregulated to promote hepatocellular carcinoma cell proliferation, probably by enhancing ATG3 and ATG7 expression	-	[94]
elongation	lncRNA HULC	epithelial ovarian carcinoma	ATG7, LC3-II, LAMP1	lncRNA HULC overexpression reduces ATG7, LC3-II, and LAMP1 expression, and then reduces apoptosis and inhibits autophagy	-	[95]

**Table 4:** Some related circRNAs at different stages of autophagy.

Autophagy Stage	circRNA	Cancer/Disease	Target	Function	Signaling Pathway/Axis	Ref.
initiation	circ_0009910	chronic myeloid leukemia	ULK1	circ_0009910 can regulate the expression of ULK1, thereby activating the level of autophagy	-	[96]
initiation	circ_CDYL	breast cancer	ATG7, ULK1	circ_CDYL regulates the expression of autophagy-related genes ATG7 and ULK1, thus promoting autophagy	-	[97]
initiation	circ_PAN3	acute myeloid leukemia	mTOR	circ-PAN3 regulates autophagy via the AMPK/mTOR signaling pathway in acute myeloid leukemia	AMPK/mTOR	[98]
initiation	circRNA ACR	RSC96 cells	mTOR	circRNA ACR in RSC96 cells promotes the activation of the PI3K/AKT/mTOR pathway to alleviate autophagy	PI3K/AKT/mTOR	[99]
initiation	circRNA ciRS-7	esophageal squamous cell carcinoma	mTOR	circRNA ciRS-7 affects the AKT–mTOR signaling pathway, thus inhibiting autophagy of ESCC cells	AKT-mTOR	[100]
vesicle nucleation	circ_MUC16	epithelial ovarian cancer	Beclin1, RUNX1, ATG13	circ_MUC16 promotes autophagy in epithelial ovarian cancer by regulating Beclin1, RUNX1, and ATG13	-	[101]
vesicle nucleation	circPOFUT1	gastric cancer	ATG12	circPOFUT1 promotes ATG12 expression to regulate autophagy-associated chemoresistance in gastric cancer	-	[102]
elongation	circ_0092276	breast cancer	ATG7, LC3-II, LC3-I, Beclin-1	circ_0092276 affects autophagy and proliferation, and represses apoptosis of breast cancer cells	-	[103]
elongation	circ_0035483	renal clear cell carcinoma cells	LC3-II, LC3-I	when circ_0035483 expression is downregulated, the LC3II/LC3I ratio is significantly reduced, thus inhibiting autophagy	-	[104]
Autophagosome formation	circ_ PABPN1	intestinal epithelial cells	ATG16L1	circ_ PABPN1 inhibits ATG16L1 translation and thus regulates autophagy in intestinal epithelial cells	-	[105]
